# Decadal Western Pacific Warm Pool Variability: A Centroid and Heat Content Study

**DOI:** 10.1038/s41598-017-13351-x

**Published:** 2017-10-13

**Authors:** Autumn Kidwell, Lu Han, Young-Heon Jo, Xiao-Hai Yan

**Affiliations:** 1 Applied Research Laboratories, The University of Texas at Austin, Austin, Texas USA; 2 College of Earth, Ocean and Environment, University of Delaware, Newark, Delaware, USA; 3 Department of Oceanography, Pusan National University, Busan, 609-735 Republic of Korea; 4 Xiamen University/University of Delaware’s Joint Institute of Coastal Research and Management, Xiamen, China

## Abstract

We examine several characteristics of the Western Pacific Warm Pool (WP) in the past thirty years of mixed interannual variability and climate change. Our study presents the three-dimensional WP centroid (WPC) movement, WP heat content anomaly (HC) and WP volume (WPV) on interannual to decadal time scales. We show the statistically significant correlation between each parameter’s interannual anomaly and the NINO 3, NINO 3.4, NINO 4, SOI, and PDO indices. The longitudinal component of the WPC is most strongly correlated with NINO 4 (R = 0.78). The depth component of the WPC has the highest correlation (R = −0.6) with NINO3.4. The WPV and NINO4 have an R-Value of −0.65. HC has the highest correlation with NINO3.4 (R = −0.52). During the study period of 1982–2014, the non-linear trends, derived from ensemble empirical mode decomposition (EEMD), show that the WPV, WP depth and HC have all increased. The WPV has increased by 14% since 1982 and the HC has increased from −1 × 10^8^ J/m^2^ in 1993 to 10 × 10^8^ J/m^2^ in 2014. While the largest variances in the latitudinal and longitudinal WPC locations are associated with annual and seasonal timescales, the largest variances in the WPV and HC are due to the multi-decadal non-linear trend.

## Introduction

Planet Earth’s entrenchment in the anthropocene is entering its third century^1,2^. Anthropogenically enhanced climate warming is the source of a prominent amount of research and concern. The current and predicted effects of a global climate warming are legion^3–5^. An important component of climate warming is the interaction between the changing climate and natural climate variability. One of the largest sources of natural climate variability is the El Niño-Southern Oscillation (ENSO).

The decadal evolution of ENSO, given its diversity and impacts, in conjunction with global warming, could be a crucial part of understanding the current and future climate dynamics. From 1980 to 2010, the intensity of El Niño events in the central Pacific Ocean almost doubled. This increase is related to the increase in intensity and frequency of central Pacific type (CP-type) El Niños since the 1990s^6^. While those results indicate that an SST warming trend in the CP region is due to the increase in CP-type El Niño activity, other research has indicated that CP-type El Niño events could occur more frequently under a projected global warming scenario^7^. The conflicting story between ENSO and climate change does not end there. Under global warming conditions, climate models predict a weakened Walker circulation and a weakened zonal thermocline gradient^8,9^. Yet, in the past three decades, observations have shown that the thermocline has steepened when CP-type El Niños are more prevalent^10,11^. While the aforementioned climate models predicted a weakened Walker circulation pattern, reanalysis data and observations yield varying results showing the equatorial winds have strengthened, weakened, or stayed the same^12–15^. The extent to which ENSO interacts with decadal-scale modulations and anthropogenic forcings in the Pacific Ocean is still unknown^16^.

A vital component of El Niño is the variability of the warm water of the tropical Pacific Ocean^17^ known as the western Pacific warm pool (WP). The WP is characterized by a mean sea surface temperature (SST) exceeding 28 °C, weak trade winds, and deep convection^18,19^. Changes of *O* (1 °C) in the SST in the WP can result in large-scale climatic change^19,20^. Satellite observations show that the SST and scale change of the WP can be related to solar irradiance variabilities, ENSO events, volcanic activities, and global warming^21^. The WP, by changing the heating and cooling of the tropical Pacific Ocean, is an important aspect of natural global climate variability with an unknown role in climate change. The heat storage of the WP is also an important variable to investigate since mechanisms for heat transfer and storage in the entire Pacific basin are often in question. While some researchers have shown mechanisms for heat transfer from the North Pacific to the tropical Pacific, others have stated that heat in the North Pacific and tropical Pacific are the result of different mechanisms of buoyancy forcing and anomalous wind forcing, respectively^22^.

We examine the role of the WP in the past thirty years. By exploring the temporal and spatial variability of the WP size and heat content, we provide a new perspective on the variability of the tropical Pacific Ocean. The analysis method involves a multi-dimensional study of the centroid migration, volume, and heat content of the WP and an application of the advanced time-series analysis technique known as Multidimensional Ensemble Empirical Mode Decomposition (MEEMD)^23,24^. We show the 30-year evolution of the warm pool during a time period of varying natural and anthropogenic climate conditions.

## Results

### Interannual Variability of the Western Pacific Warm Pool

**Figure 1:**
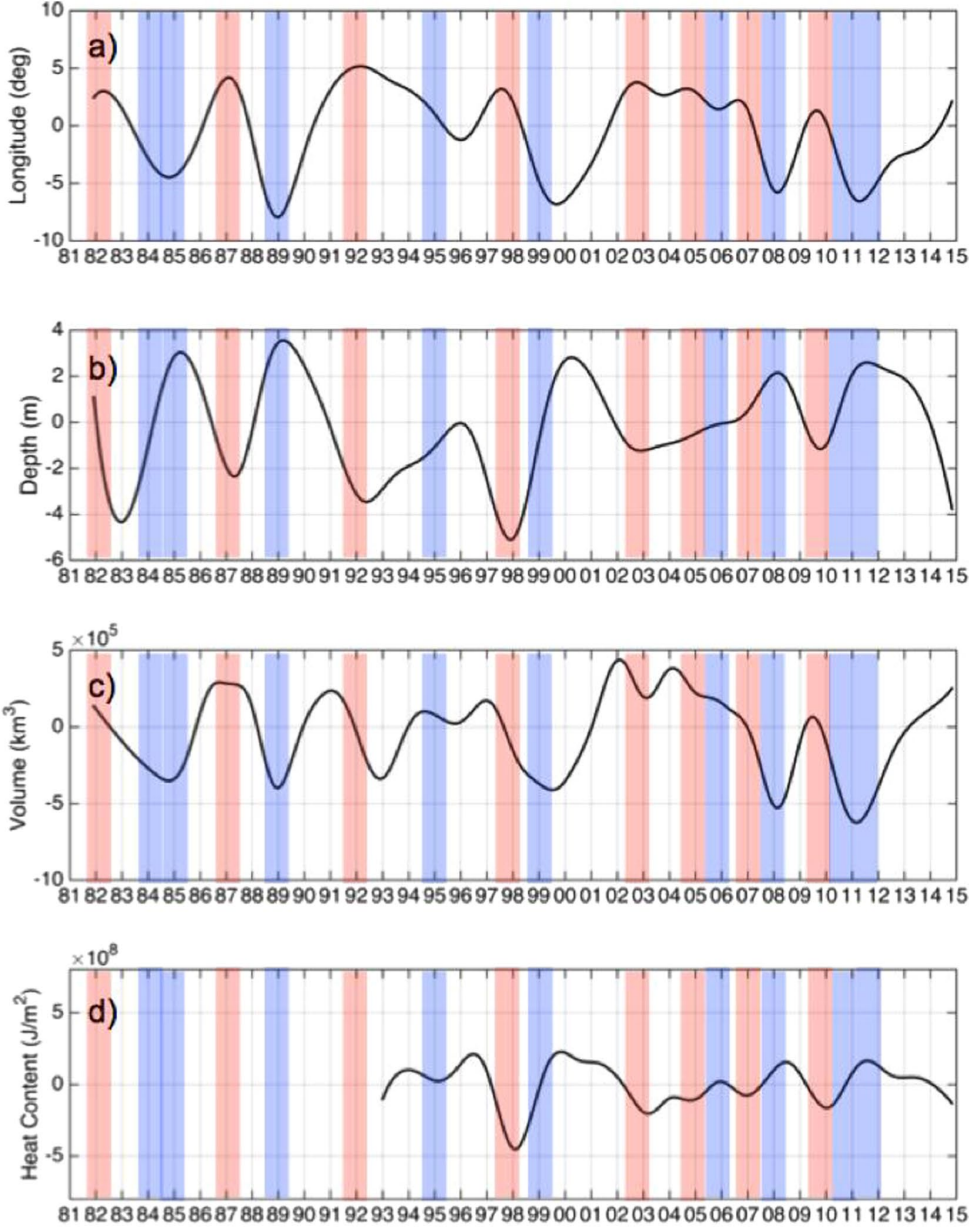
IMF 4–6 of (**a**) the WPC longitude location, (**b**) the WPC depth, (**c**) the WPV, and (**d**) the HC. El Nino years are highlighted with red and La Nina years are highlighted in blue. Created with Matlab R2016a: https://www.mathworks.com.

To determine the decadal evolution of the WP, we utilized EEMD to temporally decompose the western Pacific warm pool centroid (WPC), western Pacific warm pool volume (WPV), and the western Pacific warm pool heat content anomaly (HC) time series. (For more details about this method, please see Data and Methodology in supplementary materials). The resulting Intrinsic Mode Functions (IMFs) separate each input signal based upon their timescales in ascending order. For each decomposition, the significance of each resulting IMF was determined by calculating the spread function for the 99% and 95% confidence limit levels^25^. The energy spread of white noise can be calculated and has a Gaussian distribution with a standard deviation. For any defined confidence level, the spread lines, which bound the energy of the white noise with upper and lower limits, can be calculated. If the energy density of any IMF is above the upper spread line or below the lower spread line, then that IMF contains information at that confidence level. The results of the significance tests are shown in Table 1. The time series of interannual anomalies for the WPC, WPV, HC are shown in Fig. 1, respectively. For each time series, higher frequencies were removed from the data by EEMD. IMFs 4–6 are plotted and highlight the detrended interannual oscillations of each time series. IMFs 4–6 encompass the time scales ranging from 3 years to 30 years (Table 1). The El Niño years are highlighted in red and the La Niña years are highlighted in blue. The El Niño/La Niña classification is derived from the Oceanic Niño Index. The Oceanic Niño Index (ONI) is a standard used by NOAA to identify El Niño strength based upon the SST anomaly in the NINO 3.4 region (5°N–5°S, 120°–170 °W). [http://www.cpc.noaa.gov/products/analysis_monitoring/ensostuff/ensoyears.shtml]. From 1981 to 2014, nine El Niños and ten La Niñas occurred. While the longitudinal component of the WPC is often associated with ENSO, the interannual variability of the centroid depth, WPV and HC have not been extensively studied in this context. Figure 1b, the interannual variability of the centroid depth shows large reduction in depth during El Niños, particularly the strongest El Niño events of 1982/1983 and 1997/1998. The centroid depth is much deeper than normal during La Niña years. The WPV (Fig. 1c) exhibits large positive anomalies during most of the El Niño years and the large negative anomalies during La Nina events. The HC time series (Fig. 1d), while having an abbreviated data record starting in 1993, shows negative anomalies during El Niño events and is more positive during La Niña time periods.

**Table 1:** IMF information for each data set.

	IMF 1		IMF 2		IMF 3		IMF 4		IMF 5		IMF 6		IMF 7		Res.	
WP Parameter	var.	T	var.	T	var.	T	var.	T	var.	T	var.	T	var.	T	var.	T
Longitude Centroid	2.1	0.5	26.8	0.5	10.6	2.6	16.5	5.6	10.1	11.0	1.1	11.0	0.08+	33.0	1.2	33.0
Depth Centroid	1.6	0.3	28.4	1.0	9.3	2.5	13.8	5.5	3.4	11.0	3.4	11.0	0.2	33.0	5.0	33.0
Volume	10.3	0.5	12.8	1.0	11.3	1.0	7.5	3.7	4.5	8.3	4.1	8.3	0.3*	33.0	18.9	33.0
Heat Content	4.2	0.5	9.4	0.5	6.0	1.7	5.7	3.2	6.7	7.0	0.4	7.0	0.0*	20.0	62.9	22.0

This table shows the variance (*var*.) of each mode as a percentage of the variance of the original time series. *T* represents the peak time period of each mode in years. ^+^Denotes the variance of the mode is significant at the 95% confidence level but not the 99% confidence level. *Denotes the variance of the mode is not significant at either confidence level.

We calculated cross-correlation between the WPC longitudinal location, the WPV, WP HC, and the WPC depth. The cross-correlation measures the similarities between two time-series as a function of their lag. The leading index of the four parameters is the WPV, which leads the longitudinal migration of the WPC by 3 months. The Pacific Ocean surface wind stress^26^, a known contributor to the onset of El Niño, in the region bounded by 6°N–6°S and 180°–150°W, is well correlated with the WPV (R = −0.73) and there is no lag for the maximum correlation between the wind stress and the WPV. Interannual fluctuations in the longitudinal WPC migration lead the both the HC and WPC depth by 5 and 4 months, respectively. However, since the WPV is derived from the surface area of the WP in conjunction with the depth of the WP, then the change to the surface area of the WP is the first indicator of interannual fluctuations. The longitudinal WPC migrates with a speed of up to 25 cm/s during El Niños and over 30 cm/s during La Niñas. The WPC depth, noted as lagging behind the WPC longitude by 5 months, changes depth at the much slower pace of 200 × 10^−5^ cm/s.

To examine the linkage between WP and ENSO, we calculated the correlation coefficients and P-values of the WP centroid, WPV, and HC with various climate indices associated with El Niño (Table 2). A larger correlation coefficient (R-value) denotes a larger correlation between the time series and a smaller P-value indicates that the observed correlation is unlikely to occur by chance. The four WP parameters are all significantly correlated with several climate indices (Table 2). The longitudinal component of the WPC is most strongly correlated with NINO 4 (R = 0.78). The depth component of the WPC has the highest correlation (R = −0.6) with NINO3.4. The WPV and NINO4 have an R-Value of −0.65. HC has the highest correlation with NINO3.4 (R = −0.52). Thus, the warm pool interannual anomaly parameters (IMFs 4–6) well represent the climatic events (ENSO and PDO) that occurred during the study period.

**Table 2:** The warm pool interannual anomaly parameters (IMFs 4–6) are correlated with NINO 3, NINO 3.4, NINO 4, SOI, and PDO as shown.

No.	WP Parameter	Index	R-Value
1	Centroid Longitude	NINO 3	0.43
2	Centroid Depth	NINO 3	−0.49
3	Centroid Volume	NINO 3	0.24
4	Heat Content	NINO 3	−0.44
5	Centroid Longitude	NINO 4	0.78
6	Centroid Depth	NINO 4	−0.56
7	Centroid Volume	NINO 4	0.65
8	Heat Content	NINO 4	−0.47
9	Centroid Longitude	NINO 3.4	0.65
10	Centroid Depth	NINO 3.4	−0.6
11	Centroid Volume	NINO 3.4	0.44
12	Heat Content	NINO 3.4	−0.52
13	Centroid Longitude	SOI	−0.61
14	Centroid Depth	SOI	0.58
15	Centroid Volume	SOI	−0.38
16	Heat Content	SOI	0.45
17	Centroid Longitude	PDO	0.46
18	Centroid Depth	PDO	−0.53
19	Centroid Volume	PDO	0.26
20	Heat Content	PDO	−0.4

All R-values have an associated P-value less than 0.05 unless noted in parentheses.

### Decadal variability of the Western Pacific Warm Pool and long-term Trends

**Figure 2:**
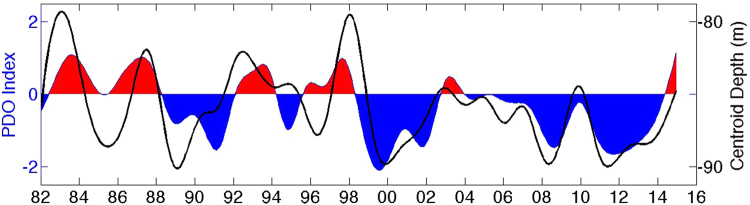
IMFs 4–6 of the WP centroid depth (*m*) are shown with a solid black line and the PDO (*unitless*) is shown shaded red (positive phase) and blue (negative phase). Created with Matlab R2016a: https://www.mathworks.com.

In this section, we discuss the relations of the WP parameters, decadal climate modes in the Pacific sector, and long-term trends. For the interannual variability, the largest variations are in association with the largest El Niño events and on longer timescales, the largest variations in the parameters are associated with the PDO. The WP parameters demonstrate a strong correlation with the PDO as shown in Table 2. From 1982 to 1998, the PDO was mostly positive. From 1999 to 2014, the PDO has been strongly negative (Fig. 2). Positive/negative PDO phases are associated with weaker/stronger trade winds in the tropical Pacific. The WP centroid depth (solid black line) and the PDO (shaded blue and red regions) are shown in Fig. 2. The WP centroid depth, the parameter with the strongest correlation with PDO (R = −0.53), has deepened from the period of 1982–1998 to 1999–2014 associated with the switch of the PDO from a positive to negative phase. However, while it might seem that the WP deepened in response to the PDO, the maximum correlation has a lag of one month, suggesting the deepening WP precedes the PDO phase. Preceding the negative PDO phase, the easterly trade wind in the tropical Pacific is stronger and WPC deepens in the water column. The stronger trade wind pushes the WP westward, leading to the more westward centroid longitude. The centroid longitude, WPV and HC are also strongly correlated with the PDO with R-values of 0.46, 0.26, and −0.4, respectively.

**Figure 3:**
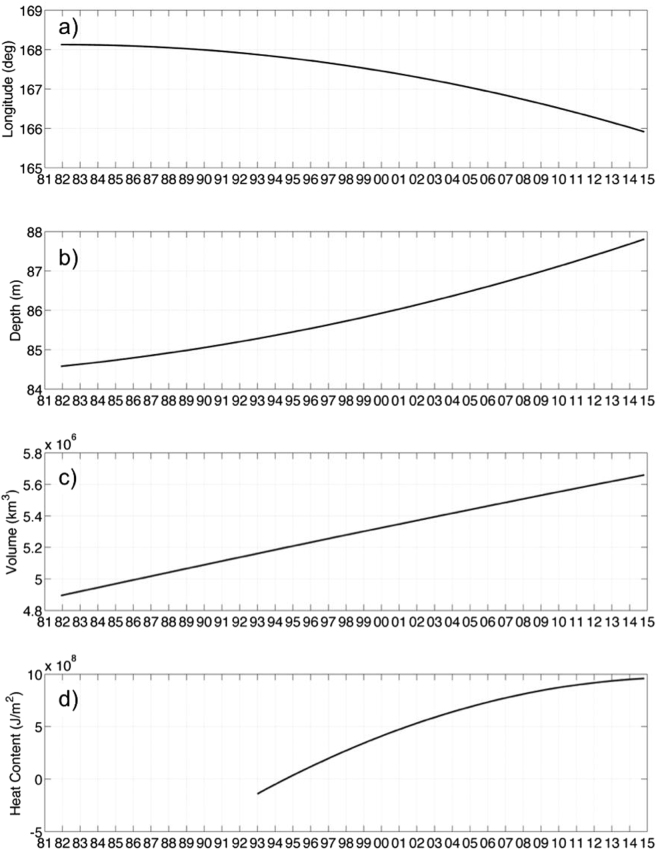
The nonlinear trends of (**a**) the WP longitudinal centroid, (**b**) the WP centroid depth, (**c**) the WPV, and (**d**) the WP HC. Created with Matlab R2016a: https://www.mathworks.com.

The residual of the EEMD represents the long-term nonlinear trend of a data set. The residuals for the WP longitudinal centroid, centroid depth, WPV, and HC are shown in Fig. 3, respectively. The WP longitudinal centroid residual (Fig. 3a) shows a westward migration toward northern Australia and Papua New Guinea. From 1982 to 2014, the longitudinal centroid location has migrated 2°. The centroid depth (Fig. 3b) increased from 84.5 m to almost 88 m. The WPV (Fig. 3c) has increased by over 700,000 km^3^, a value that is 14% larger than the initial WPV in 1982. The WP HC anomaly (Fig. 3d) slowly transitioned from a negative anomaly in the early at the beginning of the data record, to a positive anomaly in 2014. The HC anomaly increased from −1 × 10^8^ J/m^2^ to almost 10 × 10^8^ J/m^2^.

**Figure 4:**
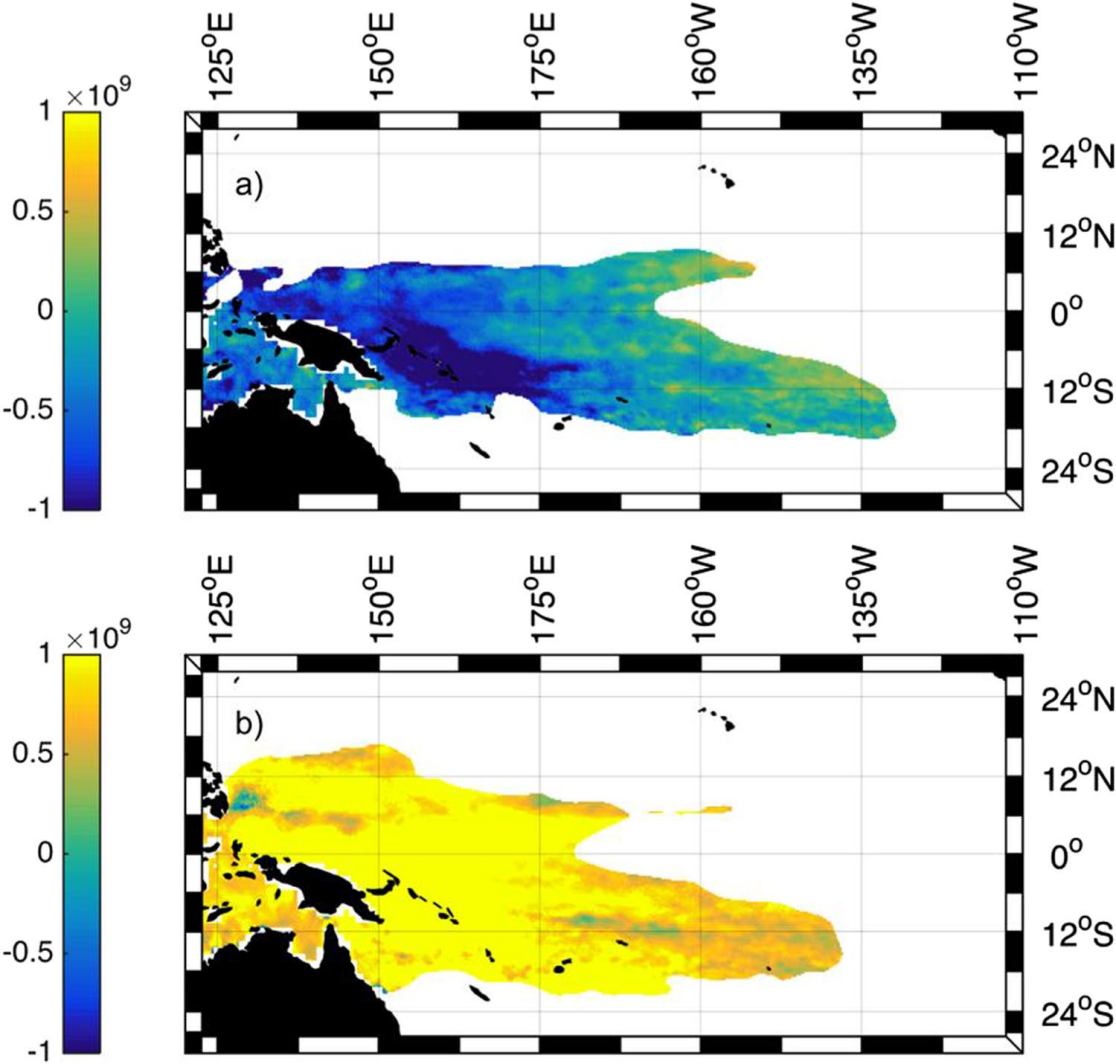
The MEEMD-derived nonlinear trend of the WP HC (J/m^2^) shown on (**a**) January 1993 and (**b**) January 2014. Created with Matlab R2016a: https://www.mathworks.com.

We also investigate spatio-temporal heat content for the WP HC during from 1993 through 2014 by utilizing MEEMD. While Fig. 3d represented the mean residual behavior for the entire WP, Fig. 4 shows the residual at each grid point within the WP. To highlight the stark differences in the warm pool from 1993 to 2014, we show the WP HC anomaly from January of 1993 (Fig. 4a) and the WP HC anomaly from January of 2014 (Fig. 4b). Both of these years are neutral ENSO years (neither El Niño or La Niña). Figure 4 shows how the warm pool has changed from a more narrow latitudinal band having large negative HC anomaly that extends to 130 °W, to a broader latitudinal band with a large positive HC anomaly that does not extend as far to the east.

## Summary and Discussion

Our study improves the understanding of the variations of WP characteristics through a systematic investigation of various WP characteristics on decadal and interdecadal time scales. In particular, we have examined the multidimensional WPC, WPV, and the WP HC. IMFs 4–6 of each parameter highlight the detrended interannual oscillations of time scales ranging from 3 years to 30 years. While the longitudinal WPC component is a much-studied aspect of ENSO, the interannual variability of the WPC depth, WPV and HC have not been extensively reviewed. The WPC depth is shallower during El Niños, particularly the strongest El Niño events of 1982/1983 and 1997/1998. The WPC depth is much deeper than normal during La Niña years. The WPV has large positive anomalies during most of the El Niño years and the large negative anomalies during La Niña events. The HC shows negative anomalies during El Niño events and is more positive during La Niña. We also investigate the time lag between the WP parameters. The WPV leads the longitudinal migration of the WPC by 3 months, and the longitudinal WPC migration leads the both the HC and WPC depth by 5 and 4 months, respectively. The WPV, as the leading mode, suggests that the change to the surface area of the WP is the first indicator of interannual fluctuations. This is further supported by the WPV’s high zero-lag correlation with the surface wind stress.

We have also reported the decadal changes of the WP associated PDO phases and climatic changes. Most notably, a deepening WPC precedes the PDO phase (Fig. 2). The WPC longitude, WPV, and HC are also strongly correlated with the PDO with R-values of 0.46, 0.26, and −0.4, respectively.

To understand the long-term nonlinear trends of the WP, we analyzed the residuals of the EEMD of the parameters and investigated the spatio-temporal residual for the HC during from 1993 through 2014 by utilizing MEEMD. The longitudinal WPC residual shows a westward migration of 2° from 1982 to 2014. The centroid depth has increased by 3.5 m, the WPV is 14% larger than the initial WPV in 1982, and the HC anomaly switched from a negative anomaly (−1 × 10^8^ J/m^2)^ in the early 90 s to a positive anomaly (10 × 10^8^ J/m^2^) in 2014. The spatio-temporal HC residual analysis shows the residual at each grid point within the WP. The warm pool has changed from a more narrow latitudinal band having large negative HC anomaly that extends to 130 °W, to a broader latitudinal band with a large positive HC anomaly restricted to the west of the Pacific Ocean near Australia and Papua New Guinea.

Several recent studies have shown that there is currently a period of global warming “hiatus” since 1998 (i.e., Easterling & Wehner 2009). The hiatus is described as a cooling of the global surface temperature^27^. However, other studies suggest that the hiatus is tied to the strongly negative PDO phase. During the negative PDO phase that began in 1998 (Fig. 2), the intensification of the Pacific trade winds resulted in the upwelling of colder water along the equator. In the past decade, a larger percentage of heat has penetrated below a 700 m depth, because of changes in the surface winds^28,29^. Our calculation of WP HC shows that even with a deepening heat storage in the Pacific Ocean, the WP is still a large source of positive heat storage anomaly. This would suggest that natural decadal climate variability plays a large role in the global surface temperatures in addition to the more commonly discussed aspects of climate change, such as sea level rise^28^. Moreover, a redistribution of heat^30,31^ is not really a global warming hiatus, and the term should be used with caution^32^. Some researchers can find no evidence of the surface warming hiatus in an updated global surface temperature analysis^33^. The long-term nonlinear trends of the WP data do not appear to be affected by the short-term negative PDO phase or the hiatus and show continually increasing or decreasing trends for the entire data record.

We studied the WP in the past thirty years of mixed interannual variability and climate change by utilizing EEMD and MEEMD for our one-dimensional and multidimensional data, respectively. By exploring the temporal and spatial variability of the WP, we provide a new perspective on the variability of the tropical Pacific Ocean. While previous studies have provided many insights on the dynamics of the WP, our study presents various WP characteristics from interannual to decadal time scales, with a novel focus on the WP HC and multidimensional centroid movement. The diagnostics of the different WP parameters described in this study can also be used as evaluation tools and to assess the reliability of projected changes associated with the WP in response to climate change.

## Data and Methods

We analyzed the past 30 years of SST data in the tropical Pacific Ocean from 1982 to 2014. The NOAA Earth System Research Laboratory Physical Sciences Division provides the SST data used in this study. The data set is an optimally interpolated SST (OISST) monthly averaged product^34^. This product combines *in situ* and satellite SST with an additional simulated SST for ice-covered regions. The temporal coverage of the data is 1981 to present. The spatial coverage is a 1.0° latitude by 1.0° longitude global grid (http://www.esrl.noaa.gov/psd/data/gridded/data.noaa.oisst.v2.html#detail).

For a three-dimensional analysis of the WP, we incorporate potential temperature profiles. The potential temperature data are from the operational ocean reanalysis system (ORAS4) implemented at European Centre for Medium-Range Weather Forecasts (ECMWF). ORAS4 is based on European Modelling of the Ocean (NEMO) V3.0 and spans the period 1958 to present. The data has a horizontal resolution 1° × 1° with equatorial refinement (0.3), 42 vertical levels, about 10 m–15 m level thickness in upper 200 m. The data is assimilated via NEMOVAR in the 3D-var FGAT mode with a 10-day assimilation window. The assimilated data includes temperature and salinity profiles from the EN3 v2a XBT bias-corrected database (1958–2009), including XBT, CTD, Argo, Mooring, and from real-time GTS thereafter, along track altimeter sea level anomalies and global trends from AVISO. SST and sea-ice are from the ERA-40 archive prior to November 1981, from the NCEP OI v2 weekly product (1981 until December 2009) and from OSTIA analysis from January 2010 onwards. The SST and sea-ice information is used to constrain the upper level ocean temperature via a newtonian relaxation scheme. Prior to 1989, the surface fluxes are from the ERA-40 atmospheric reanalysis. From 1989–2009, the surface fluxes are from ERA-Interim reanalysis. After 2010, daily surface fluxes were derived from the operational ECMWF atmospheric analysis. Data can be downloaded from: ftp://ftp.icdc.zmaw.de/EASYInit/ORA-S4/ and a full description of the data sets is available on: http://icdc.zmaw.de/easy_init_ocean.html?&L=1 ^35,36^.

The SST data and the temperature profile data are used to characterize the size and location of the WP. One method of characterizing the WP in the tropical Pacific is to track the movement of WP by assuming the warm pool can be represented as one body having an SST threshold of 28 °C^37–39^. If the WP is represented as one form, locating and tracking its centroid is a simple means of tracking the WP. The three-dimensional centroid location is determined by also incorporating a lower boundary for the WP. In our study, we use the 20 °C as the bottom boundary of the WP. The centroid location of the WP is described by Equations (1–3):

1 $$x^{\prime} =\frac{1}{n}{\sum }_{i=1}^{n}{x}_{i}$$

2 $$y^{\prime} =\frac{1}{n}{\sum }_{i=1}^{n}{y}_{i}$$

3 $$z^{\prime} =\frac{1}{n}{\sum }_{i=1}^{n}{z}_{i}$$

where *x*_*i*_ is the zonal location of a gridded location and *y*_i_ is the meridional location of a gridded location associated with SST ≥ 28 °C, and *n* represents the number of gridded locations being averaged. The centroid depth, *z*_*i*_, is ½ the depth (*d*_*i*_) of the 20 °C isotherm at the i^th^ location. The volume of the warm pool (km^3^) is calculated in Equation (4):

4 $$\begin{array}{c}y\\ 111\times 111cos(|i){d}_{i}\\ Vol={{\rm{\Sigma }}}_{i=1}^{n}\end{array}$$

where 111 × 111 cos(*y*_*i*_) represents the surface area of a gridded location associated with SST ≥ 28 °C and *d*_*i*_ is the depth of the 20 °C isotherm at the i^th^ gridded location. *n* represents the number of gridded being calculated. We also analyzed the heat content of the WP. We used the area covered by SST ≥ 28 °C as the boundaries for the WP and calculated the heat content at each grid point within that region. The sea surface height anomaly data (SSHA) used for this analysis is the Aviso Ssalto/Duacs gridded sea surface height anomalies (http://www.aviso.altimetry.fr/en/data/products/sea-surface-height-products/global.html). The temporal coverage of the data is 1992 to present. The spatial coverage is a 1/4° latitude by 1/4° longitude global grid. SSHA can be used to accurately calculate the oceanic heat storage anomalies^40,41^. The heat content anomaly is calculated in Equation (5):

5 $$\Delta H=\frac{\rho {c}_{p}}{\alpha }\Delta \eta $$

where *ΔH* is the heat storage anomaly, *Δη* is the SSHA, *ρ* is the density of seawater, *c*_*p*_ is the specific heat of seawater, and *α* is the thermal expansion coefficient of seawater. These coefficients are estimated from monthly-mean climatological data and the international thermodynamic equation of seawater^42^. The monthly-mean climatological temperature (*T*) and salinity (*S*) are available as a part of the World Ocean Atlas (https://www.nodc.noaa.gov/cgi-bin/OC5/woa13/woa13.pl?parameter=t).

The surface wind stress was calculated using the NCEP/NCAR Reanalysis-1 data provided by the NOAA/OAR/ESRL PSD, Boulder, Colorado, USA, from their Web site at http://www.esrl.noaa.gov/psd/ ^43^. The monthly means are available from 1981 to present, with a global spatial coverage of 2.5° latitude by 2.5° longitude and 17 layers of wind velocity. The wind stress was computed using the wind velocity 10 m (1000 hPa) above the surface following the method detailed by Large *et al*., (1995). The time series of the mean wind stress was computed for the region bounded by 6°N–6°S and 180°–150 °W. This is the region where interannual and multidecadal (IPO/PDO) show maximum regression on the Pacific Ocean winds^44,45^.

The main analysis method used in this study is the MEEMD mentioned in the Introduction section. MEEMD is based upon empirical mode decomposition (EMD) and ensemble empirical mode decomposition (EEMD). The purpose of EMD is to reduce a complicated one-dimensional data set into a finite and generally small number of intrinsic mode functions (IMF). This methodology empirically identifies different oscillatory modes in the data based upon their timescales and separates the data into IMFs. EMD is data-adaptive, has a high locality, and is useful for handling non-linear and non-stationary data^23,46^. EEMD improved upon the EMD method by minimizing EMD’s strong noise sensitivity, thus allowing for better physical interpretation of the IMFs^47^. MEEMD is the application of EEMD to multidimensional data and is suitable for the spatio-temporal analysis of gridded climate data^24,48^.
